# Gendered Mental Labor: A Systematic Literature Review on the Cognitive Dimension of Unpaid Work Within the Household and Childcare

**DOI:** 10.1007/s11199-023-01362-0

**Published:** 2023-04-29

**Authors:** Natalia Reich-Stiebert, Laura Froehlich, Jan-Bennet Voltmer

**Affiliations:** 1 CATALPA—Center of Advanced Technology for Assisted Learning and Predictive Analytics, Hagen, Germany; 2 Faculty of Psychology, Universitätsstr. 27, FernUniversität in Hagen, 58097 Hagen, Germany

**Keywords:** Mental labor, Cognitive load, Unpaid work, Gendered division of labor, Gender stereotypes, Social role theory, Systematic literature review

## Abstract

With this literature review, we provide a systematic overview on and working definition of mental labor in the context of unpaid work—an inherent cognitive component of daily routines primarily related to domestic or childcare tasks. Our methodology followed PRISMA guidelines, and 31 full-text articles were included. Articles were peer-reviewed and published in social science, sociological, and psychological journals. The studies applied quantitative and qualitative methodological approaches including, interviews, online surveys, observations of family routines, time estimates, and experiments. The samples covered a wide age range, consisting mostly of U.S. American or European middle-class women and men (married or in a relationship). Predominantly, the articles show that women perform the larger proportion of mental labor, especially when it comes to childcare and parenting decisions. Further, women experience more related negative consequences, such as stress, lower life and relationship satisfaction, and negative impact on their careers. We offer an integrative theoretical perspective to explain the gendered distribution of mental labor and cognitive load. We consider theoretical and practical implications of these findings for reducing gender inequality in mental labor in the context of unpaid work within the household and childcare.

The COVID-19 pandemic has produced an increase in research activity on the unequal gender distribution in unpaid work within the household and childcare, showing that families were particularly burdened with increased domestic and childcare tasks during lockdowns worldwide, and these tasks were unequally distributed between men and women, consistently to women’s detriment (e.g., Coltrane, 2000; Croda & Grossbard, 2021; Del Boca et al., 2020; Hjálmsdóttir & Bjarnadóttir, 2021; Lachance-Grzela & Bouchard, 2010; Miller, 2018; Sevilla & Smith, 2020; United Nations, 2021). Women’s greater engagement in unpaid domestic work and childcare, in turn, impedes gender equality in other domains, including workforce participation and income (e.g., Ferrant et al., 2014; Samtleben & Müller, 2022).

Research on the gendered division of unpaid work is not new (e.g., Bianchi et al., 2000, 2012). However, most studies have focused on the physical dimension of unpaid work (i.e., actually doing the housework or caring for children), whereas its cognitive dimension (i.e., thinking, planning, organizing, etc.) has received proportionally less attention. As the physical and cognitive dimensions of unpaid work are closely related (e.g., Hjálmsdóttir & Bjarnadóttir, 2021), the cognitive dimension and its impact on physical, mental, and partnership health requires more scholarly attention. For instance, some research indicates that gender inequality in mental labor might have negative implications for women’s well-being and mental health: High cognitive load or multitasking is associated with reduced capacity to exercise willpower and make long-term decisions as well as increased anxiety and stress (e.g., Daminger, 2019; Vohs et al., 2008; Wang et al., 2010; Wetherell & Carter, 2014). The fact that the cognitive dimension of unpaid work has been largely ignored in previous research might lead to an underestimation of the extent of gender inequality and its consequences in this domain. A detailed investigation of gender differences in the types and extent of mental labor in unpaid work would open up new opportunities for research and policy related to the gendered division of unpaid work (Daminger, 2019).

While unpaid work is a broad concept and can refer to activities inside or outside the household, typically, the focus is on the household and includes all responsibilities and tasks associated with maintaining a household and its family members (e.g., Ervin et al., 2022; OECD, 2021). In the current systematic literature review, we focus on mental labor concerning unpaid work in the domain of household and childcare for two main reasons. First, although mental labor might also play a role in the workplace (e.g., managing team calendars or planning team meetings; e.g., Ahn et al., 2017; Heilman & Chen, 2005), in the domain of paid work specific factors contribute to gender inequality that might not be predictive for mental labor concerning unpaid work (e.g., the gender pay gap and related gender differences in occupational and employment status; see Blau & Kahn, 2017). Second, the limited research on mental labor is mainly concerned with unpaid work, primarily with the cognitive dimension of housework and childcare.

## Defining Mental Labor

Research investigating the cognitive dimension of unpaid work in the context of household and childcare –hereafter referred to as *mental labor*– is multi-disciplinary, and various terms are used to describe it. For instance, Walzer (1996) was one of the first to coin the term mental labor and described those thinking activities accompanying the physical tasks of caring for a newborn as predominantly performed by mothers. Subsequently, in addition to the term mental work/ labor, various terms such as cognitive, mnemonic, or invisible work/ labor appeared in the literature, listing additional aspects of the cognitive dimension of unpaid work related to domestic and childcare tasks. Recently, prospective memory has also been related to mental labor (e.g., Harrington & Reese-Melancon, 2022; Niedźwieńska & Zielińska, 2021). Prospective memory refers to the memory for future actions and intentions (Einstein & McDaniel, 1990), and contains typical aspects of mental labor (e.g., intention, retention, retrieval, evaluation; see Harrington & Reese-Melancon, 2022).

Furthermore, it became apparent that some works conflate related but distinct areas of mental labor, which can be delineated thematically: First, while some definitions include an emotional component encompassing aspects of feelings and caring in partnership or family relationships (e.g., Bass, 2015; Bianchi et al., 2012; Offer, 2014; Zimmerman et al., 2002), others clearly differentiate emotion work from mental labor (e.g., Allen et al., 2008; Daminger, 2019; Hochschild & Machung, 1989; Moore, 2017). Following the latter as well as classical psychological theorizing (e.g., Forgas, 2008; Hilgard, 1980), we argue that mental labor (cognition) should be differentiated from emotion work (affect), with both being clearly distinct from the physical work of completing a task (behavior). Emotion work aims at maintaining relationships and fostering a family’s or partner’s emotional and psychological well-being (Allen et al., 2008), whereas mental labor involves the thinking and cognitive processes necessary to plan and carry out actions concerning unpaid household and childcare work.

Second, it should be noted that mental labor is often automatically accompanied by physical activities or vice versa, such as keeping a family calendar by planning appointments or writing a shopping list by remembering what needs to be bought. Often, mental labor that occurs in parallel with performing a physical activity is not only listed as a secondary outcome in the literature when gender inequalities are examined in terms of their physical aspects (e.g., Christopher, 2021; DeGroot & Vik, 2020; Faircloth, 2021; Meier et al., 2006), but also perceived as secondary by those performing mental labor (e.g., Hjálmsdóttir & Bjarnadóttir, 2021; Mederer, 1993). For this reason, it is difficult to clearly separate these two dimensions of work, which could lead to less importance being placed on the mental dimension.

Finally, we argue for differentiating mental labor from cognitive load (in the literature, the term “mental load” can also be found (e.g., Dean et al., 2022; Robertson et al., 2019), which is often used synonymously with the more common and theoretically recognized concept of cognitive load). Drawing on cognitive psychology, cognitive load can be understood as a cognitive consequence of mental labor and the associated cognitive costs of mental operations that might interfere with current task performance (Gopher, 1994). According to cognitive load theory (Sweller et al., 1998), mental efforts during learning activities occupy working memory capacity, which is inherently limited. Since only a certain amount of information can be stored and remembered in working memory, certain demands such as the intensity of cognitive processes (e.g., multitasking) increase cognitive load, which can have negative consequences such as loss of attention or errors (e.g., Lavie, 2010; Örün & Akbulut, 2019). Mental labor concerning domestic work and childcare is also characterized by cognitive multitasking (e.g., Harrington & Reese-Melancon, 2022; Offer & Schneider, 2011) and having others’ outstanding tasks on one’s mind. It can therefore limit working memory capacity and lead to cognitive load as well. Cognitive load is in turn associated with other stress-related consequences (e.g., Offer & Schneider, 2011). Thus, we argue that cognitive load is not an inherent aspect of mental labor, but a consequence thereof.

Taken together, it becomes evident that there is still no uniformly accepted definition of mental labor in the context of unpaid work. Moreover, multidisciplinary research on the different components of mental labor has led to a diverse range of findings. Therefore, the aim of this systematic review is to accumulate evidence on mental labor and to identify consistencies and differences among studies. To do so, it seeks to (a) systematize terms and definitions to provide a comprehensive working definition of mental labor, (b) summarize the different methodological approaches of the studies, including the methods and samples investigated, and (c) relate the research findings on gender differences in mental labor as well as its potential determinants and consequences. We will conclude by theoretically integrating the results of the review, discussing limitations, and identifying open questions for future research.

## Method

### Literature Search and Abstract Screening

**Fig. 1:**
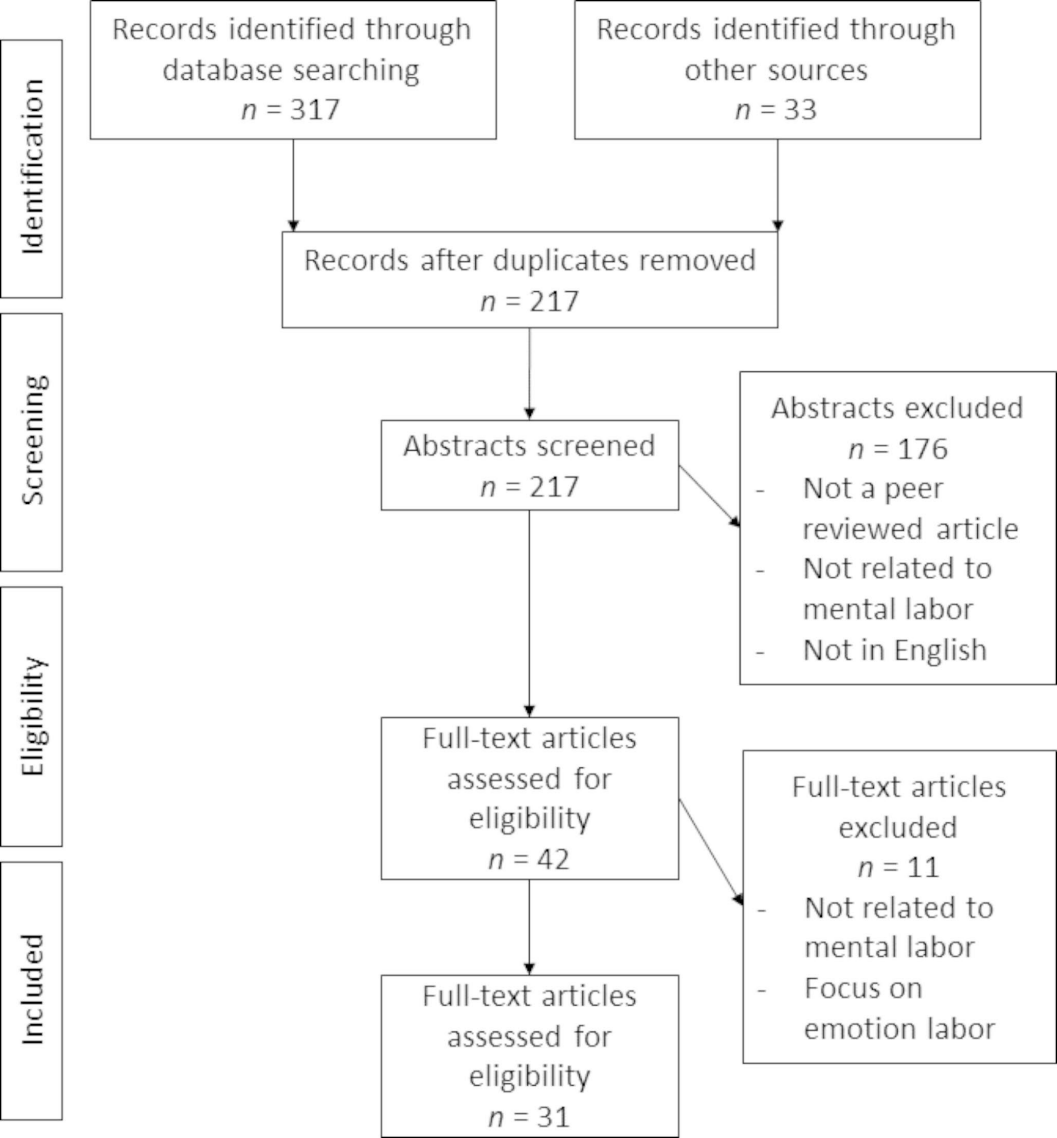
PRISMA Flow Diagram of Screening Procedures (Based on Moher et al., 2009)

We implemented the recommendations for reporting systematic reviews according to the updated Preferred Reporting Items for Systematic reviews and Meta Analyses (PRISMA, 2021) statement (Page et al., 2021), where applicable. To identify search terms, we conducted an initial search using the online search platform EBSCOhost. After an initial joint review and discussion of the extensive results, the research team members agreed to use different wordings as well as synonymous terms of mental labor. In addition, it became evident that some articles deal with mental labor in the paid work context, so we decided to include further terms that refer to unpaid work in the context of household, families, and childcare. Our final search terms were a combination of terms indicating (a) mental labor (mental labor/labour, mental work, mnemonic labor/labour, mnemonic work, cognitive labor/labour, cognitive work, invisible work, invisible labor/labour) and (b) words characterizing it as related to unpaid work (family, families, couples, dyads, household, childcare). We used these terms to search for relevant literature in titles, keywords, and abstracts listed in EBSCOhost, Scopus, and Web of Science databases in November 2021 and repeated the search in May 2022. The search was limited to peer-reviewed articles written in English, but not restricted with regards to publication date. After removing duplicates, 217 abstracts were screened by the research team (see Fig. 1).

Search results were independently screened by research team members in two steps, with inclusion and exclusion criteria established beforehand. Articles were included in the review if they (a) explicitly mentioned and dealt with mental labor or one of the synonymous terms in the context of unpaid work or (b) discussed mental labor as an additional measure alongside physical labor. Key exclusion criteria were areas to be distinguished from mental labor: (a) the mere portrayal of physical aspects of domestic work and childcare and (b) a focus on emotion work. Ambiguities about the inclusion of articles were discussed within the research team. First, titles and abstracts of the retrieved articles were screened to identify potentially relevant studies that met our inclusion criteria. Second, we consulted the full texts of the remaining articles. In addition, the reference lists of the articles were screened for further relevant articles. Thematically irrelevant articles were excluded. To reduce the risk of bias in the evaluation of the articles, the three researchers independently reviewed the selected articles and evaluated each other’s assessments. To resolve disagreements between reviewers, they were discussed in the research team.

### Data Extraction

Key contextual data were extracted separately and cross-validated by all authors. Disagreements were again resolved through a consensus process. Data extraction forms were developed by the research team and evaluated for relevance and completeness. Data included bibliographic information about the article (i.e., author(s), year of publication), sample description (i.e., sample size and characteristics), study design (i.e., methodological approach, data collection, instruments used, and analysis procedures), and main results.

## Results

### Study Approaches and Methods

Of the 31 studies included (see Table 1), 15 dealt directly with mental labor related to unpaid work and closely related topics. Sixteen studies originally addressed the physical component of unpaid work, that is, actual household and childcare tasks, and reported about mental labor as an additional outcome. Eighteen studies were qualitative, eight were quantitative survey studies, three were secondary data analyses, and two implemented experimental approaches. The qualitative studies applied the following methods: Individual interviews, joint couple interviews, focus group interviews, time recordings and diary entries, open-ended online surveys, content analyses of forum posts, and (video-recorded) observations of daily family routines. In the quantitative studies, mainly time estimates for and the distribution of mental labor as well as psychological scales were used. Several studies directly measured aspects of mental labor including thinking of household and childcare, distribution of management activities, or decision-making as well as prospective memory activities, or actions related to reminding others or relying on others to be reminded. Psychological constructs comprised partnership and life satisfaction, the perception of fairness, partnership and family conflicts, and gender attitudes. The two experimental studies examined gender differences in prospective memory involving memorization and recall of different tasks and goals.

**Table 1:** Summary of Publications Related to the Cognitive Dimension of Unpaid Work Within the Household and Childcare

Authors/ Year	Method	Sample	Procedure/ Measures	Main Results
Ahn et al., 2017	Quantitative cross-sectional survey	Study 1: *N* = 337 Study 2: *N* = 344 Study 3: *N* = 194 Study 4a: *N* = 351 Study 4b: *N* = 320 U.S. adults (18–74 years), predominantly White, on average 40% women	Closed-ended questions: - Extent to which one helps others remember their obligations and responsibilities - Extent to which one relies on others to remind one of personal obligations and responsibilities	Study 1: Providing mnemonic support to others is less typical for men. Study 2: Men are less expected to support their female partners in mnemonic work. Study 3: These lower societal expectations may result in men offering less mnemonic support to their partners. Study 4a: Mnemonic help offered by men is more likely for tasks for which they are responsible, so their help is partly self-referential. Study 4b: Corroborates study 4 through evaluations by independent raters.
Alby et al., 2014	Multimethod qualitative studies	Study 1: *N* = 15 Study 2: *N* = 8 working mothers (34–50 years) from dual-earner, middle-class families from Italy	Study 1 = semi-structured focus groups Study 2 = weekly family-related activity reports and video-recorded family interactions	Management activities are a central element of the everyday organization of a dual-income family. Related cognitive work includes planning, remembering, coordinating, and thinking ahead—activities that do not fit easily into the traditional categories of housework. Mothers were found to manage multiple, competing areas of activity (i.e., multitasking).
Bach & Aarseth, 2016*	Qualitative interview study	*N* = 22 Danish men, aged 30 to 50, upper middle-class, in partnership with ‘career women’, mostly having children	Narrative interviews	Due to women’s career orientation, housework is reorganized to involve men more. Two groups of fathers are identified: those who “run the family” and do most of the housework and care, and those who advocate an equal division of work. Reports of extensive coordination and planning to support joint functioning within personal and work life are frequently mentioned. How and by whom the planning and coordination is done is left unmentioned.
Bass, 2015	Qualitative interview study	*N* = 60 college-educated, middle- to upper-class, childless heterosexual individuals, aged 25 to 34 from the U.S. (*k* = 30 couples)	Narrative interviews conducted separately with each partner	Men are less engaged in the mental labor of anticipating parenthood. This anticipatory thinking appears to have emotional and behavioral consequences. Women are more likely to worry about combining work and family life, and to change their career goals in anticipation of the responsibilities associated with parenthood. This likely plays a key role in reproducing patterns of inequality in the labor market even before parenthood.
Christopher, 2021*	Qualitative interview study	*N* = 25 working, middle-class heterosexual couples with children from the UK	Narrative interviews while couples jointly created a household portrait discussing and assigning 25 different household tasks	In addition to the completion of domestic tasks, aspects such as planning, organization, and task assignment are mentioned. Responsibility for and management of tasks were declared by the couples as gender-specific. Both genders held women responsible for the timely and proper completion and supervision of tasks.
Ciciolla & Luthar, 2019	Quantitative cross-sectional survey	*N* = 393 U.S. married/ partnered, mostly upper-middle class mothers with dependent children at home (21–60 + years)	Measures on: - Distribution of household and childcare-related management activities - Partnership and life satisfaction	Most women indicated to have more responsibility than their partners for the mental labor related to household coordination and childcare, while responsibility for managing finances is more evenly distributed. Responsibility for household routines was associated with a feeling of being overburdened in the parenting role. Feeling solely responsible for children’s well-being was negatively related to mothers’ partnership and life satisfaction, and positively related to feelings of emptiness. Primary responsibility for household finances was negatively related to partnership satisfaction.
Coltrane, 1989*	Qualitative interview study	*N* = 20 U.S. dual-earner middle-income couples in their late thirties with children, who share childcare	Observation of families in their homes and separate interviews with fathers and mothers	Mothers were more likely than fathers to be responsible for managing and planning household tasks and childcare, with about half of couples sharing responsibility. A manager-helper dynamic was observed in some couples. The ‘helping’ husbands often waited to be told what, when, and how to complete tasks. Both mothers and fathers indicated that fathers receive more recognition for family involvement than mothers because it is naturally expected of women.
Czymara et al., 2021	Qualitative online survey	*N* = 1,119 German, highly educated, young (58% under 45), mostly female participants (78%)	Open-ended survey on psychological experiences, concerns and cognitive labor during COVID-19 lockdown	Differences concerning cognitive labor related to childcare and employment were found. Women worried more about childcare and social contacts, while men worried more about paid work during the COVID-19 lockdown.
Daly, 2002*	Qualitative interview study	*N* = 17 Canadian heterosexual dual-earner, middle-class couples in their thirties with at least one child (*k* = 50 individual and couple interviews)	In-depth, semi-structured interviews on the negotiation of family time schedules (e.g., time management, negotiation over time)	Women were primarily responsible for organizing family time and scheduling time in the household. Women took on visible parts of scheduling (e.g., writing in the family calendar, making lists) as well as its invisible aspects (e.g., worrying when things did not go as planned).
Daminger, 2019	Qualitative interview study	*N* = 70 U.S. individuals, college-educated, (upper-) middle- class, married, 25–50 years, living with at least one child under age five (*k* = 35 couples)	Separate interviews with fathers and mothers. Before interview, participants recorded household- or child-related decisions made in the last 24 h. In interview, explanatory questions on division of cognitive labor in couples’ household	Cognitive labor is classified as a four-part phenomenon consisting of anticipating needs, identifying options for meeting them, deciding between options, and monitoring outcomes. Women do a disproportionate amount of anticipation and monitoring work compared to their male counterparts. The work of decision-making is mostly collaborative. Findings suggest that inequalities in cognitive labor may be associated with relationship conflict, reduced individual well-being, and reduced job performance.
DeGroot & Vik, 2020*	Qualitative online survey	*N* = 150 U.S. mothers, predominantly married, full-time employed, college-educated, young to middle-aged, with at least one child	Open-ended questionnaire study about mothers’ responsibilities, the responsibilities of the partner, and thoughts on the (im)balance of workloads	Domestic labor and childcare are primarily the responsibility of the mother, and the partner is described as ‘helping out’. Women also report doing most of the invisible, mental work, the planning and negotiating daily work and family life (e.g., schedule play dates, plan meals, remember special occasions). Mental labor is described as a second full-time job that is exhausting, frustrating, time- and energy-consuming and not recognized by others.
Faircloth, 2021*	Longitudinal qualitative interview study	*N* = 30 mostly married, middle-aged, White, middle-class individuals in the UK (*k* = 15 dual-earner couples)	Repeated in-depth, joint and separated interviews over a five-year period about experiences becoming first-time parents	Women predominantly stated that they were responsible for the invisible labor of the thinking about managing the household. With the birth of a child, the extent of this task multiplied drastically. Even when physical tasks were equally divided, many women still reported being responsible for management and task allocation.
Forssén & Carlstedt, 2008*	Qualitative interview study	*N* = 20 Swedish women aged 63 to 83 years who mostly have cared for their (non) biological children	Repeated in-depth interviews on women’s childcare work and related health issues	The mental labor including, e.g., responsibility for childcare, constant readiness, planning activities, are described as primarily performed by women and as difficult to delineate and measure. They are often performed in parallel with other, visible activities. Mothers’ primary responsibility for childcare is assumed to pose a health risk to women.
Harrington & Reese-Melancon, 2022	Quantitative survey study	*N* = 333 U.S., mostly White mothers and fathers aged 21 to 69, fathers worked more than mothers (*n* = 149 mothers)	Several (self-developed) questionnaires, e.g.: - Prospective memory demands in parenthood - Perception of partner’s prospective memory demands - Prospective and retrospective memory	Prospective memory is identified as a form of mental labor. Regardless of gender, parents reported doing more PM tasks for their children than for their partners or themselves. Mothers reported higher PM demands than fathers, while fathers indicated PM demands were more evenly distributed. Higher PM demands for their partner were associated with more PM failure for fathers, but not for mothers. Both mothers and fathers impose greater PM demands on their partners if they believe their partner is making more PM failures.
Hjálmsdóttir & Bjarnadóttir, 2021*	Qualitative diary study	*N* = 37 highly educated, working mothers, with an average of two children, living in a relationship in Iceland	Open-ended real-time diary entries collected for two weeks (during COVID-19 pandemic) including a reflection on the day, the division of domestic tasks and responsibilities	Women reported an uneven distribution of mental labor across partners. Mothers indicated to perform the role of household management, which included planning and organizing family life and ensuring that everything works. The responsibility for dividing domestic duties mostly remained with the mothers.
Kimport, 2018	Qualitative interview study	*N* = 52 U.S. women aged 19 to 53 years, about half of whom were White and of lower-income background	Qualitative analysis of contraceptive counseling visits with clinicians	Unequal division of mental labor related to contraception with women being disproportionally responsible. The mental labor of contraception is usually viewed by clinicians as the responsibility of women. This mental labor causes emotional distress, for example, worries about the physical aspects of a contraceptive method such as side effects and associated pain.
Kincaid, 2021	Quantitative survey study	*N* = 316 U.S., employed, predominantly married mothers (21–75 years) having at least one child and working on average 38 h per week	Conducted in the framework of the General Social Survey from 2002 to 2012 Closed-ended questions on parental decision-making	Focus on parental decision-making as a component of mental labor and resulting consequences for family-to-work spillover. Maternal decision-making was marginally associated with more spillover, that is, the experience of more family-related distractions at work. Mothers, who make the majority of decisions and hold more traditional gender ideological beliefs, are more likely to experience family-to-work spillover. Mothers with a strongly egalitarian gender ideology do not differ significantly in terms of spillover from those mothers who make shared decisions with their partners. Egalitarian attitudes appear to act as a buffer against spillover associated with maternal decision-making.
Lee & Waite, 2005	Quantitative survey study	*N* = 265 married, White couples in their mid-forties with children aged 5 to 18 from the U.S. (n = 530 husbands and wives)	Data from the Sloan 500 Family Study (1999–2000) Time-use estimates (experience sampling method) including time spent thinking about household tasks	Wives and husbands spend between two and three hours per week on the mental labor of housework. However, wives spend one hour per week more on mental labor than their husbands showing that the gender gap in time spent on mental labor is comparable to the gap in time spent on physical housework. A comparison of survey and ESM estimates suggests that wives overestimate their own time, while husbands overestimate both their own and their wives’ time.
Mederer, 1993*	Quantitative survey study	*N* = 359 U.S., married, full-time employed women with at least one child (average age of youngest child = 16), husbands work more hours than wives	− Household management scale (adapted from Berk & Berk, 1979) − Estimates of time spent by respondents and spouses for task accomplishment − Further variables: e.g., perception of fairness, conflict, family demands, gender attitudes	Women perform the majority of overall household and family life management, while men are responsible for typically male-gendered and relatively infrequently performed tasks. Lower educated women and women with smaller children take on more household- and family-related management tasks. Women with traditional gender stereotypes perform more household tasks, but not management tasks. The distribution of tasks, compared to their management, shows a stronger correlation with the perception of unfairness and conflict. Management is described as a stereotypically female task for which less help is expected from men.
Meier et al., 2006*	Quantitative survey study	*N* = 45 U.S., dual-earner couples, primarily married with at least one child under age 6, mothers 19–43 years, fathers 18–56 years, women were slightly better educated	- Household management and childcare management (adapted from Mederer, 1993) - Worrying about task completion - Marital satisfaction	In terms of household management, men indicated that they share the tasks with their wives, while women indicated that they do the tasks themselves. Both partners agreed that mothers are responsible for childcare management. Performing more childcare tasks but fewer management tasks for children led to higher marital satisfaction for men. Women’s marital satisfaction was not predicted by their household or childcare-related management tasks.
Moore, 2017*	Multimethod qualitative studies	*N* = 31 mainly White, middle-class, partnered men and women with gluten-intolerant children from the U.S.	- Content analysis of posts on Facebook pages related to gluten-free families/ parenting - Interview study with *n* = 10 mothers	The mental labor related to gluten-free dieting in families is disproportionately shouldered by women. Women examined which foods were safe to eat, educated their family members, and monitored children’s eating habits. Women performed this labor even when another family member, including the child itself, could do it themselves.
Moulton-Tetlock et al., 2019	Multimethod quantitative and experimental studies	Studies 1 a-e: *N* = 465 mainly White U.S. individuals, 39% female, aged 19 to 71 Study 2: *N* = 86 mainly White U.S. individuals, aged 20 to 58 (*k* = 43 couples) Study 3: *N* = 142 U.S. individuals, 50% female, M_age_= 22.46 (*k* = 71 male– female dyads of unfamiliar individuals)	Study 1 a—e = vignettes describing stereotypically female/ male tasks for which it is to be decided whether a woman or a man should perform them Study 2 = collection of “to dos” that need to be remembered; responsibility for completion; beneficiary of completion Study 3 = experimental study on memorization of personal vs. communal goals	Examines women’s and men’s effort to prospectively remember goals that benefit others, that is, communal goals. Studies 1a-e: Most participants believed that the woman was more likely to remember communal goals and/or the man was more likely to forget them. And this not only for typically female, but also for gender-neutral tasks (except highly “male” tasks), suggesting that this type of mnemonic work may itself be gender-specific. Study 2: Women remember a greater number of communal tasks. Women’s, compared to men’s, prospective remembering has greater benefits for others. Study 3: Female participants were more likely to recall communal goals, i.e., when their partner benefited from their mnemonic work, than personal goals, where they alone benefited from their mnemonic work. Since the gender difference in memory performance occurred only for communal goals, motivational reasons and not to capacity differences between men and women are likely, which is probably due to the fact that women are socialized to care about the needs of others.
Niedźwieńska & Zielińska, 2021	Quasi-experimental study	*N* = 80 Polish individuals aged 30 to 50 years (50% female, 50% in a relationship)	Prospective memory task: sending a blank text message at a set time for seven consecutive days Covariates: e.g., number of children, time of cohabitation, education	Women showed better prospective memory performance; this gender difference was moderated by partnership status. A difference in prospective memory performance existed between women and men in a relationship, whereas no difference was found between women and men without a partner. Women who were in a relationship performed better in the prospective memory task than women who were not in a relationship, whereas men performed better when they were not in a relationship than when they were. It is hypothesized that a relationship with a male partner increases stereotypical expectations for a woman to provide mnemonic support, which contributes to better prospective memory.
Offer, 2014	Secondary data analysis	*N* = 693 parents from middle-class dual-earner, predominantly White U.S. families (*n* = 402 mothers)	Data of a subsample from the Sloan 500 Family Study - Time diary data on individuals’ experiences in a typical week - Survey data: e.g., mental labor, affect	Mothers spend about one-fourth and fathers one-fifth of their time with mental labor. Compared to men, women tend to engage more in family-specific and less in job-specific mental labor. However, gender differences were small. Mothers and fathers were equally likely to engage in family-specific mental labor during paid work and leisure activities. Family-specific mental labor was associated with lower positive and higher negative affect in mothers but not in fathers.
Offer & Schneider, 2011*	Secondary data analysis	*N* = 609 parents from middle-class dual-earner, predominantly White U.S. families *(n* = 368 mothers)	Data from the Sloan 500 Family Study - Time diary data on individuals’ primary and secondary activities (i.e., multitasking, including mental labor) - Survey data: e.g., affect, psychological distress, work-family conflict	Parents in dual-earner families spend a lot of time multitasking. There is a gender difference of about 10 h per week, with mothers multitasking more than fathers. No gender differences in terms of mental labor. For fathers and mothers, engaging in mental labor while doing something else accounts for about 8% of all multitasking tasks. Fathers are more likely to think about work-related issues, while mothers are more likely to report being pressed for time or being late. Multitasking is associated with negative affect and psychological distress for mothers. Multitasking in the company of the partner or the children is related to positive affect for both mothers and fathers.
Robertson et al., 2019	Qualitative interview study	*N* = 25 U.S. partnered mothers aged 27 to 48, with children younger than 13 years; about half not employed (*k* = 7 focus group interviews)	Phenomenological focus group interviews and semi-structured focus group protocol	The study aimed to define mental labor and its components based on the experiences of mothers of young children. Family-related mental labor is described as the thinking performed for the sake of accomplishing family goals. Six different contents of mental labor were identified. (1) planning and strategizing, (2) monitoring and anticipating needs, (3) meta parenting, (4) knowing including learning and remembering, (5) managerial thinking including delegating and instructing, and (6) self-regulation including the control of one’s own behavior and preservation of one’s own health. Invisibility and sole accountability were also described as characteristics of mental labor.
Schilperoort, 2021	Qualitative interview study	*N* = 36 individuals from church-going couples, mostly both employed, aged 20 to 80, about half with children, from New Zealand (*k* = 18 couples)	Joint in-depth interviews. Questions about responsibility for household management and who generally carries the cognitive load	Focus on religious beliefs and their relationship to gender equality in the distribution of household-related mental labor. Religious beliefs may contribute to the perpetuation of inequality in relationships. They have been identified as both facilitators and barriers to the mutual negotiation of household-related mental labor.
Treas & Tai, 2012	Quantitative international survey study	*N* = 6,871 heterosexual individuals, 18 to 65 years, married with children younger than 18 years from 31 nations	Three items measuring household management: (a) Who makes the decisions about how to raise children? (b) Who has the final say in choosing shared weekend activities? (c) Who has the final say in buying major things for the home? Covariates: age, gender, education, number of children, working hours, gender attitudes	About three-quarters of men and women make decisions about household management largely together. 23% of women, but only 2% of men, report making most parenting decisions. Married women: Are more likely to take charge of decisions about children and weekend activities when they earn more money than their husbands than when they earn less. Women’s age, education, and husbands’ work hours are positively related to the likelihood that they will be solely responsible for children and weekend activities. Married men: With respect to children, men are more likely to be the decision-makers when they take on a larger share of household chores. Age and liberal gender attitudes are negatively related to husbands’ sole responsibility related to childcare. For weekend activities, the share of housework, family income, and their liberal gender attitudes are associated with a lower likelihood of being the sole decision maker. Country differences: Compared to the egalitarian Nordic social democracies, in Latin American and Southern European countries men are more likely to take charge of the household rather than sharing decisions with their wives. Controlling for individual-level variables, in countries with more liberal gender ideologies decisions are shared rather than made by one partner. Men and women generally agree that gender-egalitarian views discourage men from sharing decisions.
Walzer, 1996	Qualitative interview study	*N* = 50 U.S. mothers and fathers aged 21 to 44 with a child under 1.5 years of age, most mothers employed (*k* = 25 couples)	Semi-structured individual and joint interviews. Questions about the experiences of men and women who have become parents.	Three categories of mental labor related to caring for a newborn: worrying, information processing, and managing the division of labor. There is a general tendency for mothers to be responsible for the mental labor of childcare, even in relationships with relatively shared physical childcare. Mothers are considered to be better informed and process more information about childcare. Accordingly, they are held responsible for its organization and implementation. Another area of mental labor for mothers is to encourage fathers to help care for their babies and delegate tasks to them. The unequal division of mental labor may be a factor in the decline in women’s marital satisfaction after the birth of the first child.
Winkler & Ireland, 2009*	Secondary data analysis	*N* = 30,032 U.S. individuals over the age of 18 (mean age in fourties), who are the reference person of their household or are the partner of the reference person	Data from the American Time Use Survey Estimates of time spent on primary and secondary household management activities (e.g., organization of and planning for household and children, activities for purchasing household, childcare, and financial services)	The average amount of time spent on household management is about 1.64 h per week. Household management is positively related to education and age, and negatively related to employment status. The results suggest a positive association between the amount of time women spend on household management and the presence of a preschool-aged child. Dual-earner wives spend significantly less time on household management than non-working wives. Household management is more evenly distributed among dual-earner couples than among couples where only the husband is employed. For dual-earner wives, having a preschool-age child is associated with more management time, but not for dual-earner husbands.
Zimmerman et al., 2002*	Qualitative interview study	*N* = 94 U.S. heterosexual, mostly White individuals (mean age in thirties) from married, dual-earner, middle-class couples having children (*k* = 47 couples)	Joint interviews (questions on strategies to manage family and work responsibilities) Combined with written questionnaires (e.g., demographic information, work, family, marital, and personality-related variables)	Most couples left family organization and household management to the wives (e.g., maintaining the family calendar, coordinating schedules). Wives are responsible for ensuring that household tasks are completed and seemed to be responsible for reminding their husbands of their family responsibilities. The couples considered this assignment of tasks to be a natural consequence of the differences in their personalities: Wives assume the role of primary organizer because their personal characteristics seem better suited for such tasks. Husbands were described as more flexible and relaxed in comparison. Women expressed no dissatisfaction with the unequal division of organizational labor.

*Note.* Studies marked with an asterisk do not primarily address the topic of mental labor, but report secondary topic-related findings.

### Samples

Across all studies, heterosexual individuals or couples were studied. Eleven studies included samples from cohabitating or married couples. Twenty studies examined individual participants, some of whom were in a relationship or married. In eight studies participants were only mothers/women, while one study investigated only men’s perspectives. Sociodemographic data (see Table 1), where available, were distributed as follows: In 15 studies, participants were young to middle-aged (18–50 years), whereas 12 studies had a wide age range (18–83 years) of participants, and one study included elder women. Twenty studies were conducted in the United States with predominantly White participants. Eight studies from Europe were conducted in Italy (1), Denmark (1), Germany (1), Poland (1), the United Kingdom (2), Sweden (1), and Iceland (1). One study was conducted in New Zealand, one in Canada, and one multinational study involved 31 countries. Twenty-one studies reported that among their participants, mothers or both partners were employed and belonged to the middle or upper class, whereas two studies involved samples with lower-class backgrounds, and four studies had samples with mixed socio-economic backgrounds. In twenty-one studies, all participants included in the samples had one or more children.

### Working Definition of Mental Labor

We classified all terms and definitions used in the articles to describe different aspects of mental labor and derived higher-level concepts of mental labor from them (see Table 2). Based on this, we propose five constitutive elements of mental labor in the context of unpaid work: *cognition*, *management*, *communal orientation, anticipation*, and *invisibility*. First, mental labor entails cognitive activities like thinking, storing and encoding information, knowing, or remembering (e.g., Ahn et al., 2017; Czymara et al., 2021; Hjálmsdóttir & Bjarnadóttir, 2021; Meier et al., 2006; Robertson et al., 2019). Second, most definitions describe mental labor as managerial, including aspects of planning, organizing, coordinating, instructing, reminding, decision-making, and monitoring (e.g., Christopher, 2021; Ciciolla & Luthar, 2019; Czymara et al., 2021; Kincaid, 2021; Offer & Schneider, 2011), with one or more individuals (e.g., one partner or both together) being responsible for these managerial tasks, keeping track of and supervising others in the completion and performance of tasks (e.g., Bach & Aarseth, 2016; Ciciolla & Luthar, 2019). Third, mental labor is described as communally oriented, that is, it is not only useful for oneself but also serves as assistance to others, is performed for the family or the partner to meet their needs and achieve communal or collective goals (e.g., Moulton-Tetlock et al., 2019; Robertson et al., 2019). Fourth, most definitions also include an anticipatory component, specifying that mental labor is often directed toward the future. Thus, it is carried out prospectively, preceding the actual performance of a task, by perceiving and anticipating potential necessities or problems (e.g., Ahn et al., 2017; Bass, 2015; Ciciolla & Luthar, 2019). Finally, some definitions include an element of invisibility in that mental labor is less tangible, difficult to detect, and often goes unnoticed by others and even by those who perform it themselves (e.g., Hjálmsdóttir & Bjarnadóttir, 2021; Mederer, 1993).

**Table 2:** Derivation of the Five Constitutive Elements of Mental Labor Based on the Terms and Definitions Used in the Articles

Terms and Definitions	Authors	Constitutive Element
- Thinking - Remembering - Knowing - Deciding - Encoding and storage of information - Process information - Retention - Recalling - Retrieval - Reflecting - Cognitive work/ labor	Ahn et al., 2017; Czymara et al., 2021; Daminger, 2019; Dean et al., 2022; Faircloth, 2021; Harrington & Reese-Melancon, 2022; Hjálmsdóttir & Bjarnadóttir, 2021; Kimport, 2018; Lee & Waite, 2005; Moulton-Tetlock et al., 2019; Niedźwieńska & Zielińska, 2021; Offer, 2014; Robertson et al., 2019; Schilperoort, 2021; Treas & Tai, 2012; Walzer, 1996	Cognition
- Planning - Organizing - Coordinating - Delegating - Monitoring - Managing - Orchestrating - Allocating tasks - Instructing - Scheduling - Maintaining control - Being responsible	Alby et al., 2014; Bach & Aarseth, 2016; Christopher, 2021; Ciciolla & Luthar, 2019; Coltrane, 1989; Czymara et al., 2021; Daly, 2002; Daminger, 2019; Dean et al., 2022; DeGroot & Vik, 2020; Faircloth, 2021; Forssén & Carlstedt, 2008; Hjálmsdóttir & Bjarnadóttir, 2021; Kimport, 2018; Kincaid, 2021; Mederer, 1993; Meier et al., 2006; Moore, 2017; Offer, 2014; Offer & Schneider, 2011; Schilperoort, 2021; Treas & Tai, 2012; Walzer, 1996; Winkler & Ireland, 2009; Zimmermann et al., 2002	Management
- Other-directed - Collective goals - Communal goals - Family needs / activities - For all in the family - Assisting others - Help partners - Provide mnemonic support for others	Ahn et al., 2017; Bass, 2015; Alby et al., 2014; Daly, 2002; Harrington & Reese-Melancon, 2022; Hjálmsdóttir & Bjarnadóttir, 2021; Kincaid, 2021; Moulton-Tetlock et al., 2019; Niedźwieńska & Zielińska, 2021; Offer, 2014; Offer & Schneider, 2011; Robertson et al., 2019	Communal orientation
- Anticipating - Thinking ahead - Foresight - Future-directed - Precedes physical work - Perceiving future problems and upcoming necessities - Prospective memorizing	Alby et al., 2014; Bass, 2015; Ciciolla & Luthar, 2019; Czymara et al., 2021; Daminger, 2019; Harrington & Reese-Melancon, 2022; Hjálmsdóttir & Bjarnadóttir, 2021; Moulton-Tetlock et al., 2019; Niedźwieńska & Zielińska, 2021	Anticipation
- Often invisible - Often goes unnoticed - Difficult to detect/ measure - Less tangible - Performed internally - Not necessarily perceived as work by the person performing it	Alby et al., 2014; Dean et al., 2022; Faircloth, 2021; Forssén & Carlstedt, 2008; Harrington & Reese-Melancon, 2022; Hjálmsdóttir & Bjarnadóttir, 2021; Mederer, 1993; Schilperoort, 2021; Walzer, 1996; Zimmermann et al., 2002	Invisibility

Integrating these aspects, we propose the following working definition: Mental labor related to unpaid work in the household and childcare is cognitive work that consists of managerial activities aimed at achieving communal goals (e.g., goals related not only to the individual, but also to the family, partner, children), which are directed toward a future outcome and goes undetected and unseen as a component of unpaid work. The primary responsibility for mental labor often lies with one individual but can also be shared.

### Gendered Division of Mental Labor 

To give a more reliable impression of the distribution of mental labor between the gender groups, only studies with samples consisting of heterosexual couples or both men and women are considered (i.e., 20 studies). The majority of these studies suggest that women are more likely to be held responsible for and perform the greater share of mental labor in the context of unpaid work, including anticipating and managing (i.e., planning, organizing, scheduling, assigning tasks to their partners, reminding, and monitoring the completion) tasks within a shared household (Ahn et al., 2017; Bass, 2015; Christopher, 2021; Coltrane, 1989; Daly, 2002; Daminger, 2019; Faircloth, 2021; Harrington & Reese-Melancon, 2022; Lee & Waite, 2005; Zimmerman et al., 2002). Three studies recorded time data and demonstrated that mental labor amounts to about two to three hours a week (Lee & Waite, 2005; Offer, 2014; Winkler & Ireland, 2009) and that women spend more of their time on mental labor than men (Lee & Waite, 2005; Offer, 2014). Four studies indicated that couples maintain a more equal distribution of mental labor in terms of household with shared responsibilities (Coltrane, 1989; Offer & Schneider, 2011) and make decisions collaboratively (Daminger, 2019; Treas & Tai, 2012). Two studies found that participants had different perceptions of whether mental labor was shared or performed exclusively by women: That is, men indicated sharing mental labor equally with their partners, while women reported that they perform it themselves (Harrington & Reese-Melancon, 2022; Meier et al., 2006). Moreover, seven studies revealed that women are more concerned about childcare as well as health issues regarding children, and make most of the parenting decisions (e.g., Czymara et al., 2021; Meier et al., 2006; Moore, 2017; Offer, 2014; Treas & Tai, 2012; Walzer, 1996; Winkler & Ireland, 2009).

### Determinants and Consequences of Mental Labor

Some articles mentioned factors that contribute to the unequal distribution of mental labor in the context of unpaid work: A central and obvious contributor lies in the *gender specificity* of this work, which –presumably socially influenced– is sex-typed as female work by both men and women (Christopher, 2021; Moulton-Tetlock et al., 2019). Women, who are stereotypically associated with communal traits (e.g., Eagly & Wood, 2012), are expected to be more concerned with this type of labor, which pursues communal goals, caregiving, and benefits others (Moulton-Tetlock et al., 2019). Accordingly, gender differences in prospective memory occur only for participants in a relationship, with women showing better recall performance than men presumably due to a higher motivation to conform to gendered expectations about communality, whereas there were no gender differences between participants not in a relationship who are not differentially motivated to remember communal goals (Niedźwieńska & Zielińska, 2021). *Occupational status* also seems to influence the distribution of mental labor: Partners who are both employed distribute mental labor more evenly, while non-employed women and women working part-time take on more mental labor than their full-time employed partners (Meier et al., 2006; Treas & Tai, 2012; Winkler & Ireland, 2009). Finally, one article suggests that (Christian) *religious beliefs* serve to perpetuate unequal patriarchal structures and expectations resulting in an unequal gendered distribution of mental labor (Schilperoort, 2021).

Potential consequences of mental labor have been examined in about one-third of the studies. Qualitative studies indicate that mental labor is described as exhausting, frustrating, and energy-consuming (DeGroot & Vik, 2020), and may lead to reduced well-being, emotional distress, and relationship satisfaction, when perceived as unequally divided between partners (e.g., Daminger, 2019; Forssén & Carlstedt, 2008; Kimport, 2018; Walzer, 1996). Moreover, mental labor in unpaid work may have an impact on paid work as (a) mental labor resulting from the anticipation of parenthood (i.e., thinking about future parenthood and the tasks and consequences associated with it) may influence women’s career choices (Bass, 2015) and (b) mental labor may lead to lower job performance due to impaired concentration on paid work tasks as mental labor tasks related to housework and childcare also pop up at the workplace, again to the disadvantage of women (Daminger, 2019; Kinkaid, 2021). Qualitative results are supported by quantitative results: unequally distributed mental labor (primarily to women’s disadvantage) and the feeling of sole responsibility for it has negative consequences. Women’s feelings of being primarily responsible for household management predicted parenting role overload, whereas feelings of being primarily responsible for child adjustment predicted lower partner and life satisfaction as well as stronger feelings of emptiness (Ciciolla & Luthar, 2019). Moreover, family-specific mental labor and multitasking at home were associated with women’s lower positive and higher negative affect as well as increased psychological distress (Offer, 2014; Offer & Schneider, 2011). Two of the articles (Daminger, 2019; Harrington & Reese-Melancon, 2022) mention cognitive load as a consequence of mental labor. Mental labor is described as time-consuming and spilling over into work and leisure time, which can create a more chronic cognitive load that interferes with performance on other tasks. Cognitive load, in turn, is described as detrimental to health and well-being.

In contrast to the results presented so far, two studies suggest that mental labor has little to no detrimental effects on women’s perceptions of unfairness and partnership satisfaction (Mederer, 1993; Meier et al., 2006). With respect to men it has been found that family-related mental labor does not influence men’s affect (Offer, 2014). Rather, fathers who do less childcare-related mental labor report higher marital satisfaction (Meier et al., 2006). Higher reliance on their partners’ mental labor, on the other hand, is associated with more forgetfulness among fathers (Harrington & Reese-Melancon, 2022). When performed jointly and simultaneously with a partner, mental labor has also been found to be associated with positive affect for both men and women (Offer & Schneider, 2011).

## Discussion

This systematic literature review focused on mental labor in the context of unpaid work, particularly in the domains of household and childcare, a topic that is receiving increased scientific attention. To the best of our knowledge, this work is the first of its kind to address the full scope of studies examining this phenomenon. Our systematic review of 31 articles makes the following major contributions to the literature: First, we described the construct with its five different dimensions (i.e., cognition, management, communal orientation, anticipation, and invisibility) and derived a conceptual definition of mental labor in the context of unpaid work. Second, we provided a comprehensive overview of the study approaches, samples, and methods used, which have been found to be interdisciplinary and multimethod. Third, our review confirmed that women perform the greater share of mental labor related to unpaid domestic work and childcare, which is associated with negative consequences for women, including emotional distress, relationship and life dissatisfaction, and career-related disadvantages.

In addition, this systematic overview has also helped us to uncover the limitations of previous research. The first limitation concerns the samples investigated in the 31 studies. Overall, heterosexual, White, well-educated, middle-class participants are overrepresented in the studies included here. These samples do not reflect the experiences of racially/ethnically or sexually diverse participants, or participants from non-Western cultural contexts. Another limitation concerns the measurement of mental labor, which is often invisible. For example, there are no validated scales for recording mental labor, which means only single facets of mental labor are measured (e.g., household management, see Meier et al., 2006). Other methods, such as time estimate studies, only capture time spent on primary activities. Mental labor, however, often runs as a secondary activity alongside a primary activity and therefore inadequately measured (e.g., Winkler & Ireland, 2009, who conclude that 2–3 h per week are spent on mental labor but assume clearly higher time investments for it).

Third, there is a lack of research investigating the association of mental labor as the cognitive dimension of unpaid work with gender differences in paid work. Concerning the physical dimension of unpaid work within the household and childcare, research has shown that women do a higher share even if both partners have similar income or the woman earns more (e.g., Syrda, 2022). Therefore, it is likely that gender differences in the cognitive dimension of unpaid work are not fully explained by gender differences in paid work (i.e., that women engage more in mental labor merely because they engage less in paid work than men), although one result of the current review was that gender differences are more pronounced when women engage in less paid work. Finally, in this emerging stream of research, many studies are mainly concerned with a description of (gender) differences in mental labor, whereas theoretical explanations for the phenomenon are not well-integrated or methodologically considered. In the remainder of this review, we thus aim to integrate multidisciplinary theoretical approaches to explain gender differences in mental labor.

### Integrating Theoretical Approaches to Mental Labor

As our multidimensional working definition shows, an explanation of gender differences in mental labor needs to incorporate multidisciplinary perspectives. In the following section, we first draw on research from cognitive psychology on prospective memory and cognitive load to describe mental labor and its consequences. We then connect these approaches with sociological and social-psychological perspectives indicating that gender differences in mental labor are likely not ability-related but motivational and rooted in social roles and gender stereotypes.

#### The Cognitive Dimension: Prospective Memory

The central aspect of most mental labor definitions is that it is a cognitive phenomenon with an anticipatory component directed toward a future event. The theoretical underpinnings for this are provided by cognitive psychology. Specifically, they are described in research on *prospective memory*. The preparatory attentional and memory process (PAM) theory (e.g., Smith, 2003; Smith & Bayen, 2004) states that preparatory attention processes require cognitive resources for successful event-based prospective memory performance. As prospective memory tasks are often performed while the individual is simultaneously engaged in other activities, successful completion of the prospective task is always to the detriment of currently ongoing tasks. Although prospective memory recall can sometimes occur effortlessly and spontaneously when the anticipated event occurs, it often requires cumbersome monitoring (Einstein & McDaniel, 2005). Constantly remembering outstanding tasks can consume working memory capacity and make it difficult to focus on other ongoing tasks (e.g., Mason & Reinholtz, 2015; Smallwood, 2011).When an appropriate situation for performing the encoded task emerges, people need to self-initiate retrieval of the goal, therefore, prospective remembering is resource-consuming in many goal pursuit situations (e.g., McDaniel et al., 2004; McDaniel & Einstein, 2000).

Research on prospective memory can be used to explain why and how mental labor can ultimately result in cognitive load when a multitude of household and childcare tasks must be remembered. Prospective memory performance is impaired when individuals have multiple memory intentions, as cognitive resources are limited, and cognitive demands eventually become too high (e.g., Anderson et al., 2019; Cohen et al., 2008; Einstein et al., 2005). Maintaining prospective memory intentions can increase cognitive load and reduce performance on other ongoing tasks (e.g., Marsh et al., 2003, 2005; Smith, 2003; Smith & Bayen, 2004). In addition to impairing performance on other tasks, cognitive load resulting from extensive mental labor is associated with further negative consequences, such as perceptions of stress and impaired psychological well-being (e.g., Ciciolla & Luthar, 2019; Daminger, 2019; Dean et al., 2022; Harrington & Reese-Melancon, 2022).

A relevant question resulting from these findings is whether gender differences in mental labor can be explained by gender differences in prospective memory ability. Converging evidence suggests that there are no substantial gender differences in prospective memory capacity (e.g., Bakker et al., 2002; Crawford et al., 2003; Efklides et al., 2002). In fact, women appear to employ more internal/ cognitive strategies like conscious rehearsal of prospective memory intentions or visualizing task performance (Niedźwieńska & Zielińska, 2021) that help them achieve higher prospective memory performance. Such strategies, in turn, have been shown to increase prospective memory performance (e.g., Kvavilashvili & Fisher, 2007; Szarras & Niedźwieńska, 2011).

Although some studies have found women to outperform men in prospective memory tasks, women seem to be more expected and more likely to take on prospective memory tasks than men. Indeed, they are more likely to be considered responsible for prospective memory tasks and to perform them more frequently than men (Ahn et al., 2017; Harrington & Reese-Melancon, 2022; Moulton-Tetlock et al., 2019). Thus, such gender differences seem to be motivational rather than ability-related (e.g., Hultsch et al., 1987; Penningroth, 2005). Therefore, to further explain gender differences in mental labor, additional aspects of our conceptual definition pertaining to the social dimension of mental labor need to be considered. To do so, we draw on social psychological and sociological research that is concerned with the individual in social context.

#### The Social Dimension: Gendered Motivation to Perform Mental Labor

Mental labor concerning household and childcare activities is an inherently social phenomenon, which is reflected in two further aspects of our conceptual definition: communal orientation and management. Mental labor, as described in the current review, concerns not only the individual performing the mental labor for themselves, but is often directed at other members of the individual’s family (i.e., partner and/or children) to achieve communal or collective goals (e.g., Moulton-Tetlock et al., 2019; Robertson et al., 2019). Moreover, the managerial aspect of mental labor also encompasses various social efforts like coordinating other individual’s tasks, instructing or reminding others about what to do, or monitoring their progress (e.g., Christopher, 2021; Ciciolla & Luthar, 2019; Czymara et al., 2021; Kincaid, 2021; Offer & Schneider, 2011). We thus conclude that mental labor is a cognitive task that is embedded in a social context.

To explain gender differences in mental labor from a social viewpoint, we draw on literature describing individual performance in collective tasks. Specifically, we argue that social psychological research on transactive memory and social loafing is relevant in explaining the motivational underpinnings of gendered mental labor. When people work on collective tasks –as is the case in the household and childcare domain in a relationship– they usually divide the cognitive work associated with the task. *Transactive memory* is the shared division of cognitive labor in relationships (e.g., Hollingshead & Fraidin, 2003) and involves the encoding, storage, retrieval, and communication of information (Wegner, 1987). The person who is expected to have the greatest knowledge of a domain is typically assigned responsibility for it (Hollingshead & Fraidin, 2003; Wegner, 1987). This touches on another aspect of our definition of mental labor, namely the responsibility that can lie with one person, but can also be shared. The relevance of a transactive memory system for mothers’ and fathers’ prospective memory performance is also discussed by Harrington and Reese-Melancon (2022). Their results showed that fathers took on less prospective memory tasks for their partner or child, which might be explained by a perception that mothers are the “experts” in prospective memory within their transactive memory system. In contrast, other work demonstrates that men are more likely to do the mental labor for stereotypically masculine tasks, e.g., men feel more responsible for managing family finances (Ciciolla & Luthar, 2019; Czymara et al., 2021; Mederer, 1993). Theoretical approaches concerning gender (in)equality offer explanations for these gender-differentiated responsibilities for prospective memory and mental labor: that is, social-constructivist perspectives and social role theory.

Social-constructivist approaches view gender as a social construct. Women and men are embedded in social contexts, relations, and family and personal processes (Allen & Hawkins, 1999). Due to these contexts and processes, they become active participants in constructing and sustaining the meaning of gender throughout implicit and explicit negotiations in family work (e.g., Coltrane, 1989; Greenstein, 1996; Thompson, 1992, 1993; West & Zimmerman, 1987). Similarly, social role theory represents a biosocial approach to gender differences in affect, cognition, and behavior (e.g., Eagly, 2009; Eagly & Wood, 2012; Wood & Eagly, 2013). Its basic premise is that gender differences are predicted by gender stereotypes, which arise from the observation of a gender division of labor in society. The observation that women and men perform different tasks (e.g., women perform household and childcare tasks more frequently than men) leads to the ascription of stereotypical traits (i.e., how men and women are and should be). Women are assumed and expected to be more communal (i.e., compassionate, nurturing) than men. Consequently, they internalize these standards and adopt communal behaviors. In turn, social roles and associated gender stereotypes lead to self-perceived attributes to conform with them (e.g., Bem, 1974; Eagly, 2009; Eagly & Crowley, 1986; Wood et al., 1997). People regulate their own behavior according to the gendered personal standards derived from social role expectations (e.g., Witt & Wood, 2010). Applied to the mental labor of unpaid work in the household and childcare, which is communal due to its social nature and orientation toward collective goals, the social expectation that women should be communal leads to the assumption that women would be more motivated to perform this labor. This argument is in line with a recent article by Niedźwieńska and Zielińska (2021), who suggest that social roles and gender stereotypes have an impact on women’s thinking and are thus relevant for explaining gender differences in mental labor. In fact, role-congruent expectations that women are more communal than men are believed to influence women’s cognition (e.g., Grysman & Hudson, 2013; Ickes et al., 2000; Klein & Hodges, 2001; Niedźwieńska, 2003). Consequently, there is a greater societal pressure for women to perform mental labor by remembering others’ goals and obligations (Ahn et al., 2017). In transactive memory tasks, the expected knowledge of the individuals involved is inferred based on gender stereotypes, making it more likely that women are assigned responsibility for communal transactive memory tasks like mental labor (Hollingshead & Fraidin, 2003). In line with this, Ciciolla and Luthar (2019) showed that mothers’ perceptions of sole responsibility for household tasks, particularly children’s adjustment, were associated with negative consequences for mothers’ well-being. The authors discuss that gender-stereotypical expectations for women to be more communal than men are associated with gendered divisions of labor not only in the physical, but also in the cognitive domain.

Social roles and gender stereotypes offer explanations not only for why women are assigned responsibility for mental labor, but also for why they are more motivated than men to invest their cognitive resources in mental labor to fulfill communal goals (Moulton-Tetlock et al., 2019). Goal congruity theory (Diekman et al., 2010) states that people pursue roles that they perceive as a good fit to their internalized values and goals. In addition, women are penalized when violating communality expectations (e.g., Eagly & Karau, 2002; Heilman & Caleo, 2018), and to avoid these backlash effects, women are motivated to fulfill communal roles in the cognitive domain as well. However, part of the costs of mental labor is that thinking about others’ outstanding needs and responsibilities reduces women’s resources to think about their own needs and responsibilities (Ahn et al., 2017).

Lastly, recent work has also explored factors explaining men’s lack of motivation to engage in communal tasks. The underrepresentation of men in communal roles stems in part from men’s perception of these roles as poorly fitting their self-concept. Because communal roles are associated with femininity, occupying these roles likely results in gender role conflict for men (e.g., Croft et al., 2015; Diekman et al., 2010) and can even lead to perceptions of lack-of-fit (Heilman & Caleo, 2018). This lack of motivation can result in social loafing, with men doing less work than women on gender-stereotypical communal tasks (Plaks & Higgins, 2000; Vancouver et al., 1991). Taken together, it is reasonable to assume that mental labor is not rooted in gender differences in respective cognitive abilities, but rather can be explained by gender differences in motivation that stem from gendered stereotypes and social expectations.

### Practical Implications and Future Research

To reduce gender inequality in domestic work and childcare (United Nations, 2015), its cognitive dimension should not be overlooked. In order to develop interventions fostering gender equality, future studies should continue to investigate the cognitive and motivational underpinnings of mental labor and its consequences. In line with the working definition provided in this review, future research on mental labor should focus on cognitive factors like prospective memory performance (i.e., cognition and anticipation), and, as the individual is situated in a social context (e.g., Ross & Nisbett, 1991), at the same time systematically examine its social particularities (i.e., management, communal orientation, invisibility). Experimental, cross-cultural and longitudinal research is needed to pinpoint the social factors associated with more or less pronounced gender stereotypes and their relations to gendered mental labor.

In relation to the interplay of cognitive and social-motivational factors of mental labor identified in our working definition, a goal of future research would be to measure the cognitive aspects and connect these to existing measures pertaining to the social particularities of gender differences identified in previous work on the physical dimension of gendered labor. To date, no encompassing scale to measure mental labor is available. Therefore, based on the working definition provided in the current research, a multi-dimensional scale to measure mental labor could be developed and psychometrically tested. Such a scale would make it easier for researchers, and in particular for couples, to grasp and assess mental labor, which can also help raise awareness of it, especially among those who primarily perform it. Second, experimental approaches to show gender differences in prospective memory and mental labor could be combined with measures of, for instance, agentic or communal self-views or social responsibility to directly show the motivational underpinnings of gendered mental labor. Third, as the gendered division of unpaid work and associated traditional gender role beliefs vary across cultures, multinational studies could relate the extent of gendered mental labor to these cultural differences and associated psychological mechanisms (e.g., traditional gender role beliefs, gendered self-views, religious or cultural values; e.g., Kosakowska-Berezecka et al., 2022).

The development of a mental labor scale would also benefit gender equality in the cognitive dimension of unpaid domestic and childcare work, as one important way to increase gender equality would be public acknowledgement and discussion, enabled by adequate operationalization and assessment of mental labor. Another major avenue would then be to foster men’s motivation to engage in communal behavior. Being communal is a trait that is generally valued positively. However, traditional gender roles inhibit prosociality in men. The GRIP (gender roles inhibiting prosociality) model (Croft et al., 2021) describes the interplay of gender role expectations and status differences that predict gender differences in prosocial behavior. Gender-inconsistent prosocial behavior (i.e., men behaving communally) can be fostered, for example, by reducing men’s fear of social backlash or by placing a higher value on communal roles and traits and strengthening the association between men and communion. Although the GRIP model is concerned with actual prosocial behaviors, we argue that the model’s tenets can also be applied to mental labor as a cognitive dimension of communal prosocial behavior.

Future research could focus on collecting larger and less selective samples and might benefit from including both partners in the relationship (i.e., cohabiting or married couples) in their samples. Studies with same-sex couples could also provide useful information about negotiating the distribution of mental labor, as traditional gender-stereotypical expectations for partners do not apply in these relationships. Moreover, studies could investigate the development of gender roles in conjunction with the motivational underpinnings of gender differences in mental labor from a developmental perspective, for instance, by assessing when and how children acquire and internalize gender-specific expectations related to the cognitive aspect of domestic work and childcare and when gender differences in mental labor manifest during childhood, adolescence, or early adulthood. Increasing awareness of the cognitive aspects of unpaid work concerning housework and childcare could strengthen existing pathways and create new avenues for promoting gender equality, for example, by engaging people –especially men– in collective action (e.g., Kosakowska-Berezecka et al., 2020). Finally, future work should focus on investigating when mental labor is perceived as too demanding and when the perception of cognitive load and related consequences manifest, as well as whether there exist intraindividual differences and predispositional determinants. Based on this, interventions should be developed that provide, for example, the affected persons themselves, counseling centers, or couple therapists with helpful advice on how to deal with mental labor and how to achieve a distribution that is perceived as fair by both partners.

## Conclusion

Gender inequality in domestic work and childcare does not only exist on the behavioral level, but also encompasses mental aspects of gender differences in socially situated cognition. This systematic literature review summarizes and integrates an emerging and promising multidisciplinary research field on mental labor with the aim of stimulating future research on the mental aspects of unpaid work within domestic tasks and childcare. We believe that current efforts to achieve gender equality, in addition to increasing awareness about this type of labor, should include a focus on fostering men’s engagement in communal social roles.

## References

[CR1] *Ahn, J. N., Haines, E. L., & Mason, M. F. (2017). Gender stereotypes and the coordination of mnemonic work within heterosexual couples: Romantic partners manage their daily to-dos. *Sex Roles*, *77*(7–8), 435–452. 10.1007/s11199-017-0743-1.

[CR2] *Alby, F., Fatigante, M., & Zucchermaglio, C. (2014). “Somebody is thinking about it”: Women as household managers in dual-earner families. *Journal of Family Research*, *26*(1), 29–48. https://doi.org/10/gqcdcm

[CR3] Allen, S. M., & Hawkins, A. J. (1999). Maternal gatekeeping: Mothers’ beliefs and behaviors that inhibit greater father involvement in family work. *Journal of Marriage and the Family*, *61*(1), 199. https://doi.org/10/c2tqc7

[CR4] Allen, S. M., Klein, S. R., & Hill, E. J. (2008). Framing carework: Context, processes and outcomes. *Journal of the Motherhood Initiative for Research and Community Involvement*. https://jarm.journals.yorku.ca/index.php/jarm/article/view/16327

[CR5] Anderson FT, Strube MJ, McDaniel MA (2019). Toward a better understanding of costs in prospective memory: A meta-analytic review. Psychological Bulletin.

[CR6] *Bach, A. S., & Aarseth, H. (2016). Adaptation, equality, and fairness. Towards a sociological understanding of ‘the supportive husband.’ *NORMA*, *11*(3), 174–189. https://doi.org/10/gqcdcj

[CR7] Bakker A, Schretlen DJ, Brandt J (2002). Testing prospective memory: Does the value of a borrowed item help people remember to get it back?. The Clinical Neuropsychologist.

[CR8] *Bass, B. C. (2015). Preparing for parenthood? Gender, aspirations, and the reproduction of labor market inequality. *Gender & Society*, *29*(3), 362–385. https://doi.org/10/f7j4bq

[CR9] Bem SL (1974). The measurement of psychological androgyny. Journal of Consulting and Clinical Psychology.

[CR10] Berk, R. A., & Berk, S. F. (1979). *Labor and leisure at home: Content and organization of the household day*. Sage Publications.

[CR11] Bianchi, S. M., Milkie, M. A., Sayer, L. C., & Robinson, J. P. (2000). Is anyone doing the housework? Trends in the gender division of household labor. *Social Forces*, *79*(1), 191. https://doi.org/10/bxbwq5

[CR12] Bianchi, S. M., Sayer, L. C., Milkie, M. A., & Robinson, J. P. (2012). Housework: Who did, does or will do it, and how much does it matter? *Social Forces*, *91*(1), 55–63. https://doi.org/10/ggfnn7 10.1093/sf/sos120 PMC4242525 25429165

[CR13] Blau FD, Kahn LM (2017). The gender wage gap: Extent, trends, and explanations. Journal of Economic Literature.

[CR14] *Christopher, E. (2021). Capturing conflicting accounts of domestic labour: The household portrait as a methodology. *Sociological Research Online*, *26*(3), 451–468. https://doi.org/10/gqcdck

[CR15] *Ciciolla, L., & Luthar, S. S. (2019). Invisible household labor and ramifications for adjustment: Mothers as captains of households. *Sex Roles*, *81*(7–8), 467–486. https://doi.org/10/gm8s77 10.1007/s11199-018-1001-x PMC8223758 34177072

[CR16] Cohen AL, Jaudas A, Gollwitzer PM (2008). Number of cues influences the cost of remembering to remember. Memory & Cognition.

[CR17] Coltrane, S. (1989). Household labor and the routine production of gender. *Social Problems*, *36*(5), 473–490. https://doi.org/10/gfscq5

[CR18] *Coltrane, S. (2000). Research on household labor: Modeling and measuring the social embeddedness of routine family work. *Journal of Marriage and Family*, *62*(4), 1208–1233. 10.1111/j.1741-3737.2000.01208.x.

[CR19] Crawford J, Smith G, Maylor E, Della Sala S, Logie R (2003). The prospective and retrospective memory questionnaire (PRMQ): Normative data and latent structure in a large non-clinical sample. Memory (Hove, England).

[CR20] Croda, E., & Grossbard, S. (2021). Women pay the price of COVID-19 more than men. *Review of Economics of the Household*, *19*(1), 1–9. https://doi.org/10/gptv4c 10.1007/s11150-021-09549-8 PMC7883943 33613141

[CR21] Croft A, Atkinson C, Sandstrom G, Orbell S, Aknin L (2021). Loosening the GRIP (gender roles inhibiting prosociality) to promote gender equality. Personality and Social Psychology Review.

[CR22] Croft A, Schmader T, Block K (2015). An underexamined inequality: Cultural and psychological barriers to men’s engagement with communal roles. Personality and Social Psychology Review.

[CR23] *Czymara, C. S., Langenkamp, A., & Cano, T. (2021). Cause for concerns: Gender inequality in experiencing the COVID-19 lockdown in Germany. *European Societies*, *23*(sup1), 68–81. 10.1080/14616696.2020.1808692.

[CR24] *Daly, K. (2002). Time, gender, and the negotiation of family schedules. *Symbolic Interaction*, *25*(3), 323–342. 10.1525/si.2002.25.3.323.

[CR25] *Daminger, A. (2019). The cognitive dimension of household labor. *American Sociological Review*, *84*(4), 609–633. https://doi.org/10/ggpcbh

[CR26] Dean, L., Churchill, B., & Ruppanner, L. (2022). The mental load: Building a deeper theoretical understanding of how cognitive and emotional labor overload women and mothers. *Community, Work & Family*, *25*(1), 13–29. https://doi.org/10/gqcdcd

[CR27] *DeGroot, J. M., & Vik, T. A. (2020). The weight of our household rests on my shoulders”: Inequity in family work. *Journal of Family Issues*, *41*(8), 1258–1281. 10.1177/0192513X19887767.

[CR28] Del Boca, D., Oggero, N., Profeta, P., & Rossi, M. (2020). Women’s and men’s work, housework and childcare, before and during COVID-19. *Review of Economics of the Household*, *18*(4), 1001–1017. https://doi.org/10/ghcrnv 10.1007/s11150-020-09502-1 PMC7474798 32922242

[CR29] Diekman AB, Brown ER, Johnston AM, Clark EK (2010). Seeking congruity between goals and roles: A new look at why women opt out of Science, Technology, Engineering, and Mathematics careers. Psychological Science.

[CR30] Eagly, A. H. (2009). The his and hers of prosocial behavior: An examination of the social psychology of gender. *American Psychologist*, *64*(8), 644–658. https://doi.org/10/b84qgs 10.1037/0003-066X.64.8.644 19899859

[CR31] Eagly AH, Crowley M (1986). Gender and helping behavior: A meta-analytic review of the social psychological literature. Psychological Bulletin.

[CR32] Eagly AH, Karau SJ (2002). Role congruity theory of prejudice toward female leaders. Psychological Review.

[CR33] Eagly, A. H., & Wood, W. (2012). Social role theory. In P. Van Lange, A. Kruglanski, & E. Higgins, *Handbook of theories of social psychology* (pp. 458–476). Sage Publications. 10.4135/9781446249222.n49

[CR34] Efklides A, Yiultsi E, Kangellidou T, Kounti F, Dina F, Tsolaki M (2002). Wechsler memory scale, rivermead behavioral memory test, and Everyday Memory Questionnaire in healthy adults and Alzheimer patients. European Journal of Psychological Assessment.

[CR35] Einstein, G. O., & McDaniel, M. A. (1990). Normal aging and prospective memory. *Journal of Experimental Psychology: Learning, Memory, and Cognition*, *16*(4), 717–726. https://doi.org/10/dtszvh 10.1037//0278-7393.16.4.717 2142956

[CR36] Einstein GO, McDaniel MA (2005). Prospective memory: Multiple retrieval processes. Current Directions in Psychological Science.

[CR37] Einstein GO, McDaniel MA, Thomas R, Mayfield S, Shank H, Morrisette N, Breneiser J (2005). Multiple processes in prospective memory retrieval: Factors determining monitoring versus spontaneous retrieval. Journal of Experimental Psychology: General.

[CR38] Ervin J, Taouk Y, Alfonzo LF, Hewitt B, King T (2022). Gender differences in the association between unpaid labour and mental health in employed adults: A systematic review. The Lancet Public Health.

[CR39] *Faircloth, C. (2021). When equal partners become unequal parents: Couple relationships and intensive parenting culture. *Families Relationships and Societies*, *10*(2), 231–248. 10.1332/204674319X15761552010506.

[CR40] Ferrant, G., Pesando, L. M., & Nowacka, K. (2014). *Unpaid care work: The missing link in the analysis of gender gaps in labour outcomes*. OECD Development Centre. https://www.oecd.org/dev/development-gender/Unpaid_care_work.pdf.

[CR41] Forgas JP (2008). Affect and cognition. Perspectives on Psychological Science.

[CR42] *Forssén, A. S. K., & Carlstedt, G. (2008). “You really do something useful with Kids”: Mothering and experienced health and illness in a group of elderly Swedish women. *Health Care for Women International*, *29*(10), 1019–1039. https://doi.org/10/bgd2vq 10.1080/07399330802269683 18821212

[CR43] Gopher, D. (1994). Analysis and measurement of mental load. In P. Bertelson, P. Eelen, & G. d’Ydewalle (Eds.), *International perspectives on psychological science, Vol. 2. The state of the art* (pp. 265–291). Lawrence Erlbaum Associates, Inc.

[CR44] Greenstein TN (1996). Husbands’ participation in domestic labor: Interactive effects of wives’ and husbands’ gender ideologies. Journal of Marriage and the Family.

[CR45] Grysman A, Hudson JA (2013). Gender differences in autobiographical memory: Developmental and methodological considerations. Developmental Review.

[CR46] *Harrington, E. E., & Reese-Melancon, C. (2022). Who is responsible for remembering? Everyday prospective memory demands in parenthood. *Sex Roles*, *86*(3–4), 189–207. 10.1007/s11199-021-01264-z.

[CR47] Heilman ME, Caleo S (2018). Combatting gender discrimination: A lack of fit framework. Group Processes & Intergroup Relations.

[CR48] Heilman ME, Chen JJ (2005). Same behavior, different consequences: Reactions to men’s and women’s altruistic citizenship behavior. Journal of Applied Psychology.

[CR49] Hilgard, E. R. (1980). The trilogy of mind: Cognition, affection, and conation. *Journal of the History of the Behavioral Sciences*, *16*(2), 107–117. 10.1002/1520-6696(198004)16:2<107::AID-JHBS2300160202>3.0.CO;2-Y. 10.1002/1520-6696(198004)16:2<107::aid-jhbs2300160202>3.0.co;2-y 11608381

[CR50] *Hjálmsdóttir, A., & Bjarnadóttir, V. S. (2021). I have turned into a foreman here at home”: Families and work–life balance in times of COVID-19 in a gender equality paradise. *Gender Work & Organization*, *28*(1), 268–283. 10.1111/gwao.12552. 10.1111/gwao.12552 PMC7537149 33041540

[CR51] Hochschild, A., & Machung, A. (1989). *The second shift: Working parents and the revolution at home*. Viking.

[CR52] Hollingshead AB, Fraidin SN (2003). Gender stereotypes and assumptions about expertise in transactive memory. Journal of Experimental Social Psychology.

[CR53] Hultsch DF, Hertzog C, Dixon RA (1987). Age differences in metamemory: Resolving the inconsistencies. Canadian Journal of Psychology/Revue Canadienne de Psychologie.

[CR54] Ickes W, Gesn PR, Graham T (2000). Gender differences in empathic accuracy: Differential ability or differential motivation?. Personal Relationships.

[CR55] *Kimport, K. (2018). More than a physical burden: Women’s emotional and mental work in preventing pregnancy. *Journal of Sex Research*, *55*(9), 1096–1105. https://doi.org/10/gqcdch 10.1080/00224499.2017.1311834 PMC6115298 28418714

[CR56] *Kincaid, R. (2021). Maternal decision-making and family-to-work spillover: Does gender ideology matter? *Sociological Focus*, *54*(3), 223–238. https://doi.org/10/gqcdcg

[CR57] Klein KJK, Hodges SD (2001). Gender differences, motivation, and empathic accuracy: When it pays to understand. Personality and Social Psychology Bulletin.

[CR58] Kosakowska-Berezecka, N., Besta, T., Bosson, J. K., Jurek, P., Vandello, J. A., Best, D. L., Wlodarczyk, A., Safdar, S., Zawisza, M., Żadkowska, M., Sobiecki, J., Agyemang, C. B., Akbaş, G., Ammirati, S., Anderson, J., Anjum, G., Aruta, J. J. B. R., Ashraf, M., & Bakaitytė, A., … & Žukauskienė, R. (2020). Country‐level and individual‐level predictors of men’s support for gender equality in 42 countries. *European Journal of Social Psychology*, *50*(6), 1276–1291. 10.1002/ejsp.2696

[CR59] Kosakowska-Berezecka, N., Bosson, J. K., Jurek, P., Besta, T., Olech, M., Vandello, J. A., Bender, M., Dandy, J., Hoorens, V., Jasinskaja-Lahti, I., Mankowski, E., Venäläinen, S., Abuhamdeh, S., Agyemang, C. B., Akbaş, G., Albayrak-Aydemir, N., Ammirati, S., Anderson, J., & Anjum, G., … & Żadkowska, M. (2022). Gendered self-views across 62 countries: A Test of competing models. *Social Psychological and Personality Science*, 194855062211296. 10.1177/19485506221129687

[CR60] Kvavilashvili L, Fisher L (2007). Is time-based prospective remembering mediated by self-initiated rehearsals? Role of incidental cues, ongoing activity, age, and motivation. Journal of Experimental Psychology: General.

[CR61] Lachance-Grzela M, Bouchard G (2010). Why do women do the lion’s share of housework? A decade of research. Sex Roles.

[CR62] Lavie, N. (2010). Attention, distraction, and cognitive control under load. *Current Directions in Psychological Science*, *19*(3), 143–148. https://doi.org/10/frdp8v

[CR63] *Lee, Y. S., & Waite, L. J. (2005). Husbands’ and wives’ time spent on housework: A comparison of measures. *Journal of Marriage and Family*, *67*(2), 328–336. 10.1111/j.0022-2445.2005.00119.x.

[CR64] Marsh RL, Hicks JL, Cook GI (2005). On the relationship between effort toward an ongoing task and cue detection in event-based prospective memory. Journal of Experimental Psychology: Learning Memory and Cognition.

[CR65] Marsh RL, Hicks JL, Cook GI, Hansen JS, Pallos AL (2003). Interference to ongoing activities covaries with the characteristics of an event-based intention. Journal of Experimental Psychology: Learning Memory and Cognition.

[CR66] Mason MF, Reinholtz N (2015). Avenues down which a self-reminding mind can wander. Motivation Science.

[CR67] McDaniel MA, Einstein GO (2000). Strategic and automatic processes in prospective memory retrieval: A multiprocess framework. Applied Cognitive Psychology.

[CR68] McDaniel MA, Guynn MJ, Einstein GO, Breneiser J (2004). Cue-focused and reflexive-associative processes in prospective memory retrieval. Journal of Experimental Psychology: Learning Memory and Cognition.

[CR69] *Mederer, H. J. (1993). Division of labor in two-earner homes: Task accomplishment versus household management as critical variables in perceptions about family work. *Journal of Marriage and the Family*, *55*(1), 133. 10.2307/352964.

[CR70] *Meier, J. A., McNaughton-Cassill, M., & Lynch, M. (2006). The management of household and childcare tasks and relationship satisfaction in dual-earner families. *Marriage & Family Review*, *40*(2–3), 61–88. 10.1300/J002v40n02_04.

[CR71] Miller T (2018). Paternal and maternal gatekeeping? Choreographing care. Sociologica.

[CR72] Moher, D., Liberati, A., Tetzlaff, J., Altman, D. G., & Group, T. P. (2009). Preferred reporting items for systematic reviews and meta-analyses: The PRISMA statement. *PLOS Medicine*, *6*(7), Article e1000097. https://doi.org/10/bq3jpc PMC3090117 21603045

[CR73] *Moore, L. R. (2017). Food intolerant family: Gender and the maintenance of children’s gluten-free diets. *Food Culture & Society*, *20*(3), 463–483. 10.1080/15528014.2017.1288792.

[CR74] *Moulton-Tetlock, E. E., Ahn, J. N., Haines, E. L., & Mason, M. F. (2019). Women’s work: Remembering communal goals. *Motivation Science*, *5*(2), 157–178. https://doi.org/10/gm8s74

[CR75] Niedźwieńska A (2003). Gender differences in vivid memories. Sex Roles.

[CR76] *Niedźwieńska, A., & Zielińska, M. (2021). Gender differences in remembering about things to do depend on partnership status. *Sex Roles*, *84*(3), 139–151. 10.1007/s11199-020-01158-6.

[CR77] OECD (2021). *Caregiving in crisis: Gender inequality in paid and unpaid work during COVID-19*. https://www.oecd.org/coronavirus/policy-responses/caregiving-in-crisis-gender-inequality-in-paid-and-unpaid-work-during-covid-19-3555d164/

[CR78] *Offer, S. (2014). The costs of thinking about work and family: Mental labor, work–family spillover, and gender inequality among parents in dual-earner families. *Sociological Forum*, *29*(4), 916–936. https://doi.org/10/f6shgg

[CR79] *Offer, S., & Schneider, B. (2011). Revisiting the gender gap in time-use patterns: Multitasking and well-being among mothers and fathers in dual-earner families. *American Sociological Review*, *76*(6), 809–833. 10.1177/0003122411425170.

[CR80] Örün, Ö., & Akbulut, Y. (2019). Effect of multitasking, physical environment and electroencephalography use on cognitive load and retention. *Computers in Human Behavior*, *92*, 216–229. https://doi.org/10/ghvpjx

[CR81] Page, M. J., McKenzie, J. E., Bossuyt, P. M., Boutron, I., Hoffmann, T. C., Mulrow, C. D., Shamseer, L., Tetzlaff, J. M., Akl, E. A., Brennan, S. E., Chou, R., Glanville, J., Grimshaw, J. M., Hróbjartsson, A., Lalu, M. M., Li, T., Loder, E. W., Mayo-Wilson, E., McDonald, S., & Moher, D. (2021). The PRISMA 2020 statement: An updated guideline for reporting systematic reviews. *BMJ*, n71. https://doi.org/10/gjkq9b 10.1136/bmj.n71 PMC8005924 33782057

[CR82] Penningroth S (2005). Free recall of everyday retrospective and prospective memories: The intention-superiority effect is moderated by action versus state orientation and by gender. Memory (Hove, England).

[CR83] Plaks JE, Higgins ET (2000). Pragmatic use of stereotyping in teamwork: Social loafing and compensation as a function of inferred partner–situation fit. Journal of Personality and Social Psychology.

[CR84] *Robertson, L. G., Anderson, T. L., Hall, M. E. L., & Kim, C. L. (2019). Mothers and mental labor: A phenomenological focus group study of family-related thinking work. *Psychology of Women Quarterly*, *43*(2), 184–200. https://doi.org/10/gjt4nv

[CR85] Ross, L., & Nisbett, R. E. (1991). *The person and the situation: Perspectives of social psychology*. Pinter & Martin Ltd.

[CR86] Samtleben C, Müller KU (2022). Care and careers: Gender (in)equality in unpaid care, housework and employment. Research in Social Stratification and Mobility.

[CR87] *Schilperoort, L. (2021). Striving towards equal partnerships: Church-going couples and the division of household-related mental labour. *New Zealand Sociology*, *36*(1), 77–101. 10.3316/informit.850297028557490.

[CR88] Sevilla, A., & Smith, S. (2020). Baby steps: The gender division of childcare during the COVID-19 pandemic. *Oxford Review of Economic Policy*, *36*(Supplement_1), 169–186. https://doi.org/10/gmcrhq 10.1093/oxrep/graa027 PMC7499756 40504206

[CR89] Smallwood J (2011). The footprints of a wandering mind: Further examination of the time course of an attentional lapse. Cognitive Neuroscience.

[CR90] Smith RE (2003). The cost of remembering to remember in event-based prospective memory: Investigating the capacity demands of delayed intention performance. Journal of Experimental Psychology: Learning Memory and Cognition.

[CR91] Smith RE, Bayen UJ (2004). A multinomial model of event-based prospective memory. Journal of Experimental Psychology: Learning Memory and Cognition.

[CR92] Sweller, J., van Merrienboer, J. J. G., & Paas, F. G. W. C. (1998). Cognitive architecture and instructional design. *Educational Psychology Review*, *10*(3), 251–296. https://doi.org/10/fxd3d5

[CR93] Syrda, J. (2022). Gendered housework: Spousal relative income, parenthood and traditional gender identity norms. *Work Employment and Society*, 095001702110697. 10.1177/09500170211069780.

[CR94] Szarras K, Niedźwieńska A (2011). The role of rehearsals in self-generated prospective memory tasks. International Journal of Psychology.

[CR95] Thompson L (1992). Feminist methodology for family studies. Journal of Marriage and the Family.

[CR96] Thompson L (1993). Conceptualizing gender in marriage: The case of marital care. Journal of Marriage and the Family.

[CR97] *Treas, J., & Tai, T. (2012). How couples manage the household: Work and power in cross-national perspective. *Journal of Family Issues*, *33*(8), 1088–1116. 10.1177/0192513X11426700.

[CR98] United Nations (2015). *Transforming our world: The 2030 agenda for sustainable development*. https://sdgs.un.org/2030agenda

[CR99] United Nations (2021). *The sustainable development goals report*. https://unstats.un.org/sdgs/report/2021/

[CR100] Vancouver JB, Rubin B, Kerr NL (1991). Sex composition of groups and member motivation III: Motivational losses at a feminine task. Basic and Applied Social Psychology.

[CR101] Vohs KD, Baumeister RF, Schmeichel BJ, Twenge JM, Nelson NM, Tice DM (2008). Making choices impairs subsequent self-control: A limited-resource account of decision making, self-regulation, and active initiative. Journal of Personality and Social Psychology.

[CR102] *Walzer, S. (1996). Thinking about the baby: Gender and divisions of infant care. *Social Problems*, *43*(2), 219–234. https://doi.org/10/gjt4nz

[CR103] Wang J, Novemsky N, Dhar R, Baumeister RF (2010). Trade-offs and depletion in choice. Journal of Marketing Research.

[CR104] Wegner, D. M. (1987). Transactive memory: A contemporary analysis of the group mind. In B. Mullen & G. R. Goethals (Eds.), *Theories of group behavior* (pp. 185–208). Springer. 10.1007/978-1-4612-4634-3_9

[CR105] West C, Zimmerman DH (1987). Doing gender. Gender & Society.

[CR106] Wetherell MA, Carter K (2014). The multitasking framework: The effects of increasing workload on acute psychobiological stress reactivity. Stress and Health.

[CR107] *Winkler, A. E., & Ireland, T. R. (2009). Time spent in household management: Evidence and implications. *Journal of Family and Economic Issues*, *30*(3), 293–304. https://doi.org/10/cxs56j

[CR108] Witt MG, Wood W (2010). Self-regulation of gendered behavior in everyday life. Sex Roles.

[CR109] Wood W, Christensen PN, Hebl MR, Rothgerber H (1997). Conformity to sex-typed norms, affect, and the self-concept. Journal of Personality and Social Psychology.

[CR110] Wood W, Eagly AH (2013). Biology or culture alone cannot account for human sex differences and similarities. Psychological Inquiry.

[CR111] *Zimmerman, T. S., Haddock, S. A., Ziemba, S., & Rust, A. (2002). Family organizational labor: Who’s calling the plays? *Journal of Feminist Family Therapy*, *13*(2–3), 65–90. 10.1300/J086v13n02_05.

